# Stability Formulation for Integrated Opto-mechanic Phase Shifters

**DOI:** 10.1038/s41598-018-20405-1

**Published:** 2018-01-31

**Authors:** Yigit Ozer, Serdar Kocaman

**Affiliations:** Electrical and Electronics Engineering Department, Middle East Technical University, Ankara, 06800 Turkey

## Abstract

Stability of opto-mechanical phase shifters consisting of waveguides and non-signal carrying control beams is investigated thoroughly and a formula determining the physical limitations has been proposed. Suggested formulation is not only beneficial to determine physical strength of the system but also advantageous to guess the response of the output to the fabrication errors. In the iterative analysis of cantilever and double-clamped beam geometrical configurations, the stability condition is revealed under the strong inter-dependence of the system parameters such as input power, device length and waveguide separation. Numerical calculations involving effective index modifications and opto-mechanic movements show that well-known cantilever beams are unstable and inadequate to generate φ = 180° phase difference, while double-clamped beam structures can be utilized to build functional devices. Ideal operation conditions are also presented in terms of both the device durability and the controllability of phase evolution.

## Introduction

Integration of many photonic devices such as modulators, phase shifters and interferometers on the same chip^1,2^ is a prominent way to enhance performance of new generation communication^3^ and quantum information processing^4,5^ technologies as well as quantum data storage^6^ techniques. Main motivation of these systems comes from the minimization of power consumption and coupling losses for faster, accurate and compact implementations^7,8^. Recent advancements show that further developments in on-chip communication links with the help of the nano-scaled structures provided by the cutting-edge lithographic capabilities will keep being initiated. Moreover, compatibility with complementary metal-oxide-semiconductor (CMOS) technology offers accurate fabrication of nano-mechanical devices including movable parts to enhance capabilities of these systems with the help of broadband opto-mechanical operation characteristics^9–14^.

For the on-chip photonic network configurations, phase shifter^9,15,16^ is an essential part of the network and the required phase shift of light field can be achieved with a variety of different methods. Although change in the device length results in an optical path length difference and shifts the phase of the light, it is clearly not practical for on-chip applications. Instead, most commonly used method is to inject carriers into the waveguide by using a p-i-n diode to change effective index under the control of bias voltage^17–21^. Alternatively, the phase of the signal can be controlled by temperature dependent nature of effective index^22–24^. However, thermal-optical effect phase shifters suffer from high power consumption as well as the long response time. In addition, closely placed thermo-optical devices result in thermal crosstalk even in room temperature^25^. Another type of phase shifter uses the motion of resonant structures to rearrange resonant frequency^26–31^. However, these methods generate practical difficulties as well since all of them provide desirable performance only in narrowband.

In addition to the methods mentioned above, the other prominent way of manipulating the effective index in a broad spectrum for the integrated devices is to utilize two optically coupled waveguides by varying the distance between them^9,10,32–35^ and the method for controlling this distance determines the characteristics of the device operation. The capacitive actuator is the conventional micro electro-mechanical system (MEMS) based device and the effective index change is provided from the conversion of the electrical signal into a mechanical movement^32,35^. These structures require two metalized contact layers, which are desired to be placed far away from waveguides in order to limit the absorption of the light in the metal. Therefore, the system has an important risk of suffering from metal-induced optical losses^35^ and relatively larger footprint. Necessity of electrodes may not introduce a problematic issue on single device architectures; however, it is clearly a limiting factor for the complex photonic on-chip products with many optical elements.

Furthermore, required optical force to adjust the position of the waveguides can also be generated by the laterally coupled light between signal and control waveguides without additional voltage signal^36^. With this method, both attractive and repulsive forces can be obtained depending on the modes being in-phase and out-of-phase inside the coupled waveguides, respectively^36,37^. As the intensity of coupled field increases mainly in the near field, this force is more significant for nano-scale devices. In addition, unlike capacitive actuators, physical footprint could be quite small and there is no metallic component that can initiate optical losses. Therefore, all optical phase shifters^9,10,38–41^ can be more advantageous for scalable on-chip applications. Moreover, typically, the separation between optical beams for capacitive devices is relatively large (~250 nm)^32,35^, and hence required deflection for the perfect phase shift operation is greater than the all optical devices, making the power requirement higher. For instance, MEMS based devices usually need 10 V (corresponds to 7 W optical power for a previously reported structure^32^) or more applied potential^32,35^ as an input signal. In comparison, ~8–17 mW laser power may provide similar performance in the opto-mechanical (all-optical) components as thoroughly explained in the following sections.

Opto-mechanical studies have seemed to focus on deformable cantilever and double-clamped beam structures^9,10,40,41^ for light force-driven devices to reach broadband phase shift of φ = 180°. Here, considering the fact that durability of these systems is an important benchmark to build reliable devices, stability conditions have been investigated for these structures and a mathematical modal has been proposed to reveal device behavior covering many input variations. Performance of these devices has been also analyzed in terms of the phase generation and controllability.

## Results

### Physical Device

**Figure 1:**
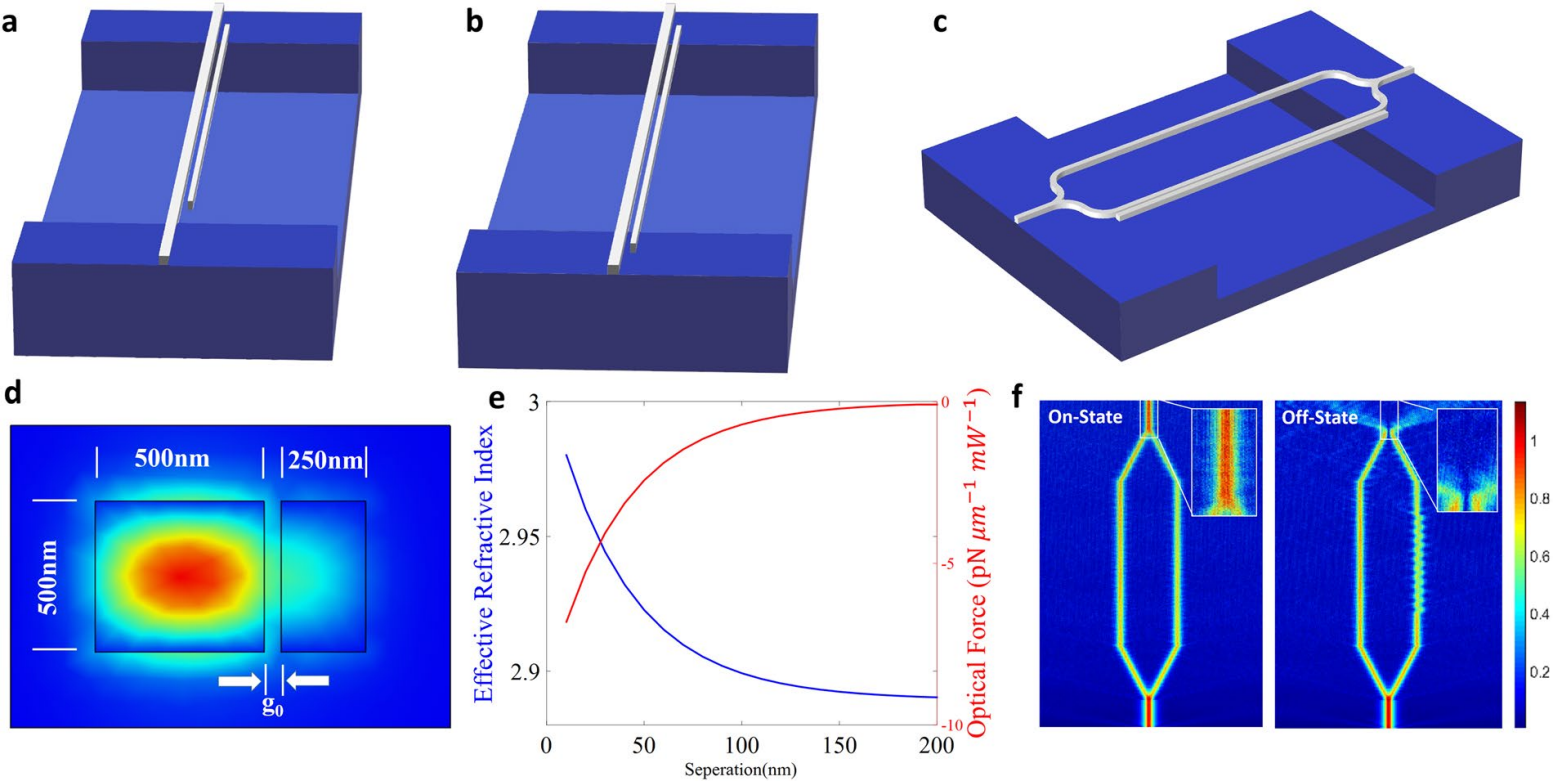
Illustration of the utilized phase shifter structures. The signal carrying waveguide and the control beam are placed parallel to each other. (**a**) The control waveguide is a cantilever beam where one end is free. (**b**) The non-signal-carrying waveguide is a double-clamped beam with fixed ends. (**c**) Projection of all optical modulator design using the Mark-Zehnder interferometer (MZI), where the opto-mechanical phase shifter is placed one side of the MZI. (**d**) Modal distribution of field in cross-sectional view. The field intensity is illustrated with colors from red (high intensity) to dark blue (low intensity). (**e**) Effective index and optical force density with respect to the separation. (**f**) Beam propagation method (BPM) simulation result (representative) of MZI output showing both on-state and off-state of the modulator. The insets show zoom of the field intensity at the output.

The usual opto-mechanic phase shifter structure consists of one waveguide and one non-signal carrying control beam, which is either a cantilever or a double-clamped beam and placed parallel to the signal carrying waveguide^9,10,40,41^. While one end of the cantilever beam is free, the double-clamped and signal carrying waveguides are fixed in both ends as shown in Fig. 1a and b, respectively.

The main idea is to insert the phase shifter into a Mach Zehnder Interferometer (MZI) as shown in Fig. 1c so that the shifted phase can be utilized to control the output intensity in modulator or switching applications. The height and width of waveguide are both 500 nm where beam width is 250 nm since narrower waveguide design is more convenient in terms of a higher deflection (Fig. 1d). While effective index values of this system are calculated by using Finite Element Method (FEM), Euler-Bernoulli beam theory^42^ is utilized for the precise calculation of deflection as a result of optical gradient force. Figure 1e shows that this force creates a continuous load to the beams and if the separation is more than a certain value (100 nm in this geometry), the force does not change considerably by the beam movement. As it is shown below in more details, deflection values (~30 nm, here) that could provide π phase shift (Fig. 1f) modifies the optical gradient force significantly along the waveguide. Therefore, due to this strong inter-dependence between the deflection and the force, an iterative solution method should be applied for the set of equations describing this system (see Methods section). In each iteration, force and deflection are reevaluated using previous values until the force variations along the waveguide are converged to a steady state value.

**Figure 2:**
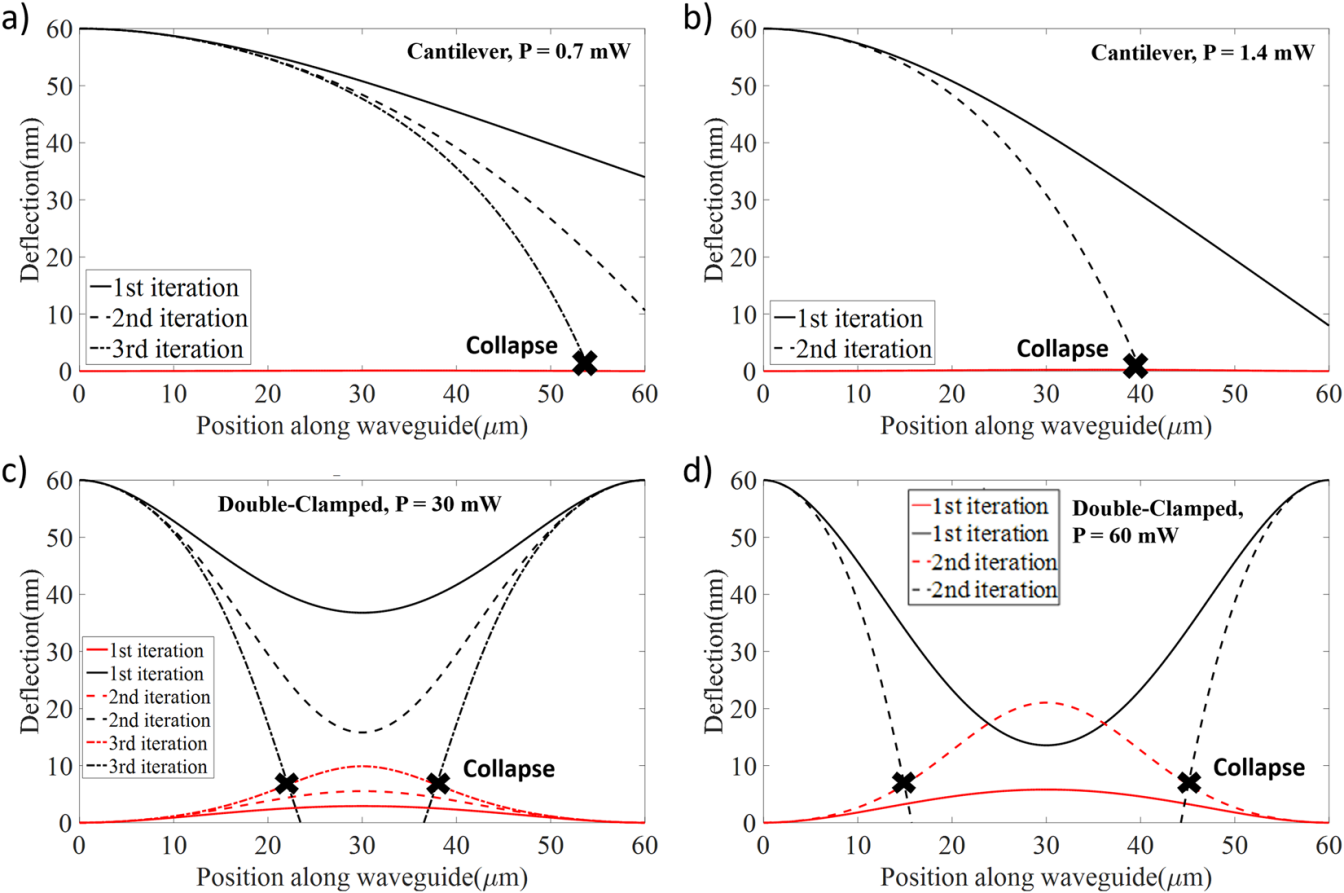
Deflection curves for the waveguide (red) and the control beam (black) with respect to the consecutive iterations and collapse points. Solid lines represent the deflections as they were modeled in the previous studies where the optical force is assumed to be independent of displacement, while dashed lines represent the results of the iterations solutions. Crosses point out the collapse points. The highly deflected appearance in the waveguides is due to the fact that X-axis is in µm scale and Y-axis is in nm scale. (**a,b)** Deflection curve of cantilever beam structures (*g* = 60 nm; *l* = 60 µm) in case input power is 0.7 mW and 1.4 mW. (**c,d)** Deflection curve of double-clamped beam structures (*g* = 60 nm; *l* = 60 µm) for the input power of 30 mW and 60 mW.

Figure 2 summarizes the analysis showing the interdependence between the deflection and the optical forces for both structures with 60 nm initial separation. For the cantilever case, free-end is deflected by ~26 nm and ~52 nm as shown in Fig. 2a,b for the 0.7 mW and 1.4 mW launched powers, respectively. Once these deflection are taken into account (for the second iteration), the cantilever beam which is exposed to 1.4 mW actually collapses. In addition, collusion also occurs for the structure with 0.7 mW after the third iteration. Similarly, double-clamped beams (Fig. 2c,d) generate ~23.2 nm and ~46.6 nm deflections (for 30 mW and 60 mW launched powers) in the middle of control waveguide initially, and with rapidly increasing displacement of the beams, physical contact happens in a few iterations as seen in Fig. 2c,d. These results are critically important since the structures and the power levels are quite similar with the previously proposed silicon devices as the potential phase shifters^9,10^ and the iterative method explained above clearly shows that the stability of the system can only be determined only after several iterations. Consequently, estimating the breakdown condition and the possible maximum phase shift with a particular structure become crucial since the motivation is to use these implementations as phase shifters.

**Figure 3:**
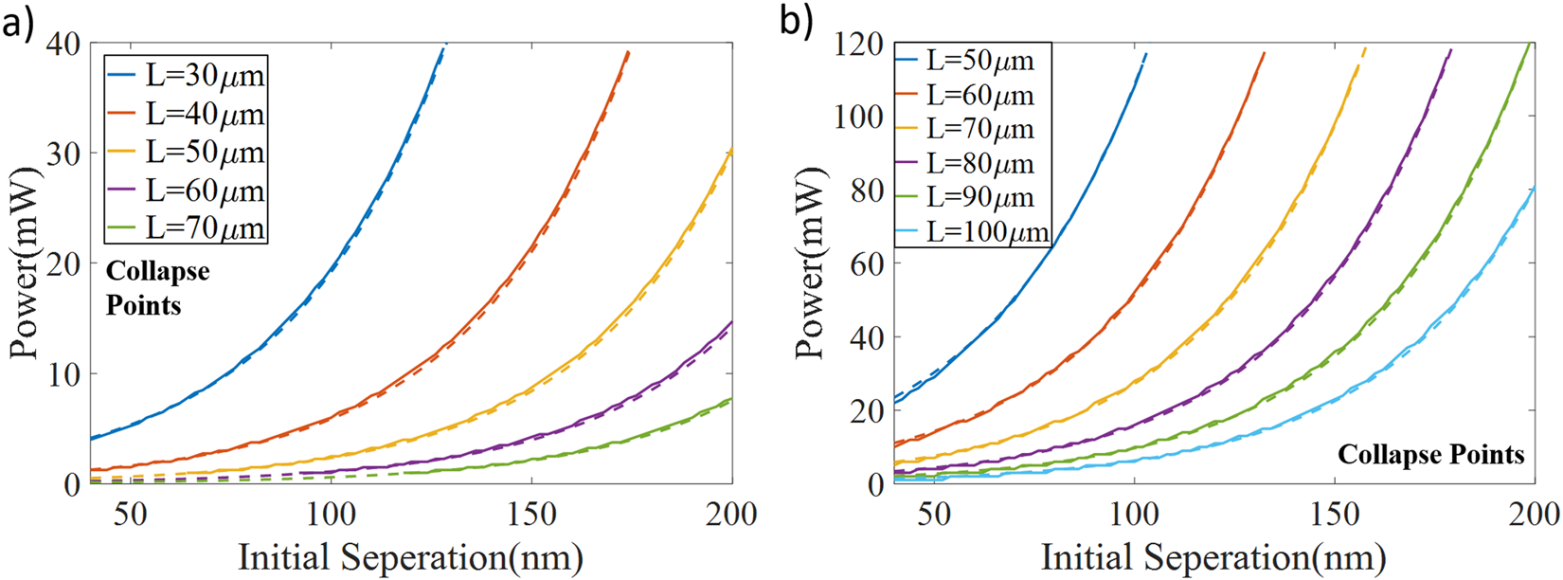
Collapse points for various phase shifter designs. Actual break points are represented with solid lines and evaluated points are shown as dashed lines for (**a)** cantilever and (**b)** double-clamped beams. The region under each curve represents the conditions for physically stable system.

In order to investigate these critical points further, a number of numerical simulations have been performed. In the calculations presented here, devices are assumed to be in steady state if the displacement has not changed more than 0.025 nm between two iterations, which means calculations have been performed up to the tenth iteration for the precise displacement and phase values. Within the scope of these simulations, launched power has been slowly increased until the collapse happens for a given waveguide geometry (similar to the structures in the previous studies^9,10^) and the maximum power that the system can handle is noted. These simulations have been calculated for both cantilever and double clamped structures where the initial separation between the main waveguide and nano-beam providing the phase control has been scanned between 40 nm and 200 nm for a variety of different control waveguide length values. Figure 3a represents the results for cantilever case and Fig. 3b shows these conditions for the double clamped structure. There are a couple of quite interesting observations in these results and the first one is about the relation between the maximum power noted and the initial separation. For both structures and for all the device geometries, the maximum power for which the system can stay stable seems to be exponentially increasing with higher separation between waveguides. This exponential dependency can be explained from the similar relation between optical force and separation (see Methods). The other important observation is about the relation between the physical length of the coupling waveguide and again the maximum power that the system can withstand without being broken. Interestingly, waveguide becomes quickly more fragile as the length of the waveguide increases, for the same separation for both structures.

Quantifying the possible maximum power with respect to the geometrical parameters for a particular design could be useful in terms of the system stability especially when there is a precise design parameter and fabrication errors are desired to be taken into account without going through the relatively tedious FEM simulations. For this purpose, the data obtained from the numerical calculations above has been mathematically fitted and the formula below has been proposed for the maximum power that the system can handle with given initial waveguide separation and waveguide length.

1 $$P=A\frac{\exp (Bg)}{{L}^{C}}$$

Here, P (mW) is the maximum power level that the system is still stable, g (nm) defines the initial separation between waveguides, L (µm) represents the control waveguide length (interaction length), B and C are the constants governing the initial separation and the device length dependencies, respectively and A is a general proportionality constant. For the waveguide structures above, $B=0.255$ and $C=4.109$ for both structures where *A* =2.93 × 10^10^ for cantilever beam and *A* =1.3584 × 10^12^ for double-clamped beam cases. Results for the proposed formulation have also been presented in the Fig. 3a and b where the solid lines are the maximum power values obtained from the simulations and dashed lines are from the formula above. There is clearly an almost perfect match between the proposed formulation results and numerical collapse conditions for both cantilever and double-clamped devices.

In addition to the waveguide structure studied above, another geometry (height = 1 µm, signal waveguide width = 400 nm, control waveguide width = 200 nm) which is taken exactly from a previous study^10^ has also been analyzed and the same values have been obtained for B and C together with updated A values (see Supplementary Information). Therefore, the proposed formulation is indeed quite effective in obtaining the system stability condition and having the same values for B and C constants for different geometries (and also phase shifter designs - cantilever or double-clamped) proves the robustness of the formulation in terms of covering the aimed dependency. Moreover, proposed formulation can also provide the opportunity to predict effect of aberrations in the actual device dimensions due to the current lithography challenges (i.e. to guess the response of the system to the fabrication errors).

### Phase Generation

Next, the accumulated phase difference has been studied in detail, as the purpose of the studied devices here is to design integrated phase shifters. Phase difference generated due to the opto-mechanical motion of the control waveguide can be calculated by dividing the interaction region between waveguides into small intervals and integrating over the interaction length (Equation 2).

2 $$\phi =\int \frac{2\pi }{\lambda }({n}_{eff}(z)-{n}_{eff}(0))dz$$

**Figure 4:**
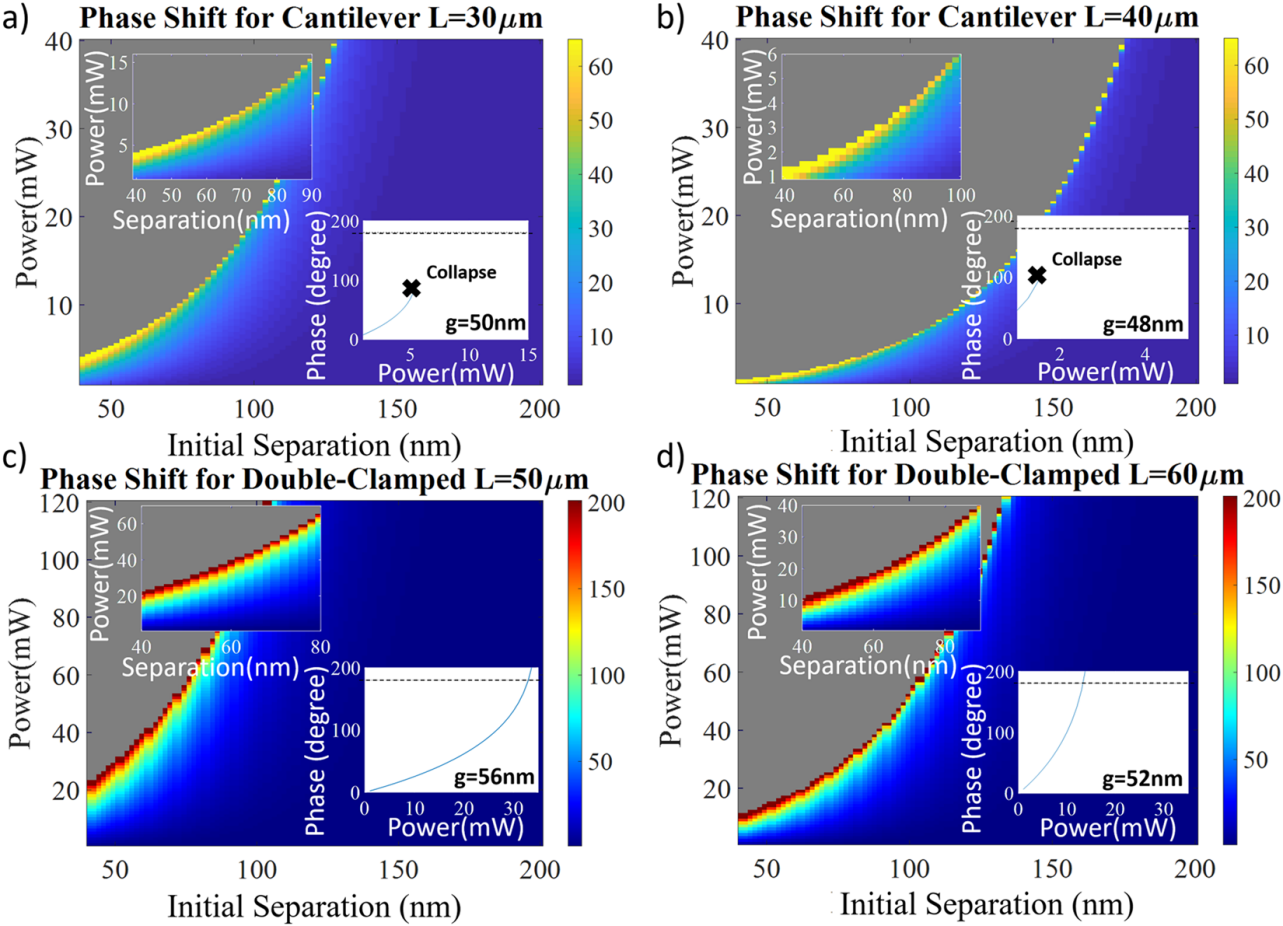
Generated phase shift (in color) with respect to the power and initial separation values. Unstable conditions are represented in gray color. The insets show zoom of the ideal operation regions and generated phase values with respect to the input power for specified initial separation. (**a,b)** Phase generation of cantilever beam structures for 30 µm and 40 µm of device lengths. (**c,d)** Generated phase difference for double-clamped beams of 50 µm and 60 µm device lengths.

These calculations have been performed within the system’s stability region (i.e. within the possible maximum power limits) with the help of the proposed formulation above by using intervals of ~2 nm. Both cantilever and double-clamped based structures have been studied by varying the geometrical parameters and the results have been summarized in Fig. 4.

The obvious observation for the Fig. 4 is that there is no significant phase change generated for most of the stable region and the sizable amount of phase change is introduced just before the system collapses. Since smaller separation increases the optical force and this elevated force decreases the separation even further, getting a quicker mechanical movement (and more effective index change leading to a higher phase difference) before the gap between the waveguides closes is indeed logical. This sensitive balance shows one more time the need for a precise mathematical model that can predict the consequences of geometrical variations coming from fabrication errors in these phase shifters. In addition, easily deformable nature of cantilever leads to a more fragile balance than the case for the double-clamped structure. As a result, both maximum utilizable power and maximum phase generation are lower than the ones for double-clamped beam structures. More importantly, cantilever beams cannot maintain the physical state for high deflections since a small power variation results in a significant displacement even at a modest phase change value (~60°). This situation is so severe that cantilever beams always collapse before they can generate significant phase shift (Fig. 4a,b). This means, the required phase shift for a modulator (φ = 180°) is not even numerically achievable for silicon-based devices. Therefore, combining this inability with the fact that sensitivity to the power variations indicates that cantilever structures may not be efficiently used as opto-mechanical phase shifters. On the other hand, double-clamped structures are able to generate more than π phase difference with relatively less dependency on the power variations (Fig. 4c,d).

**Figure 5:**
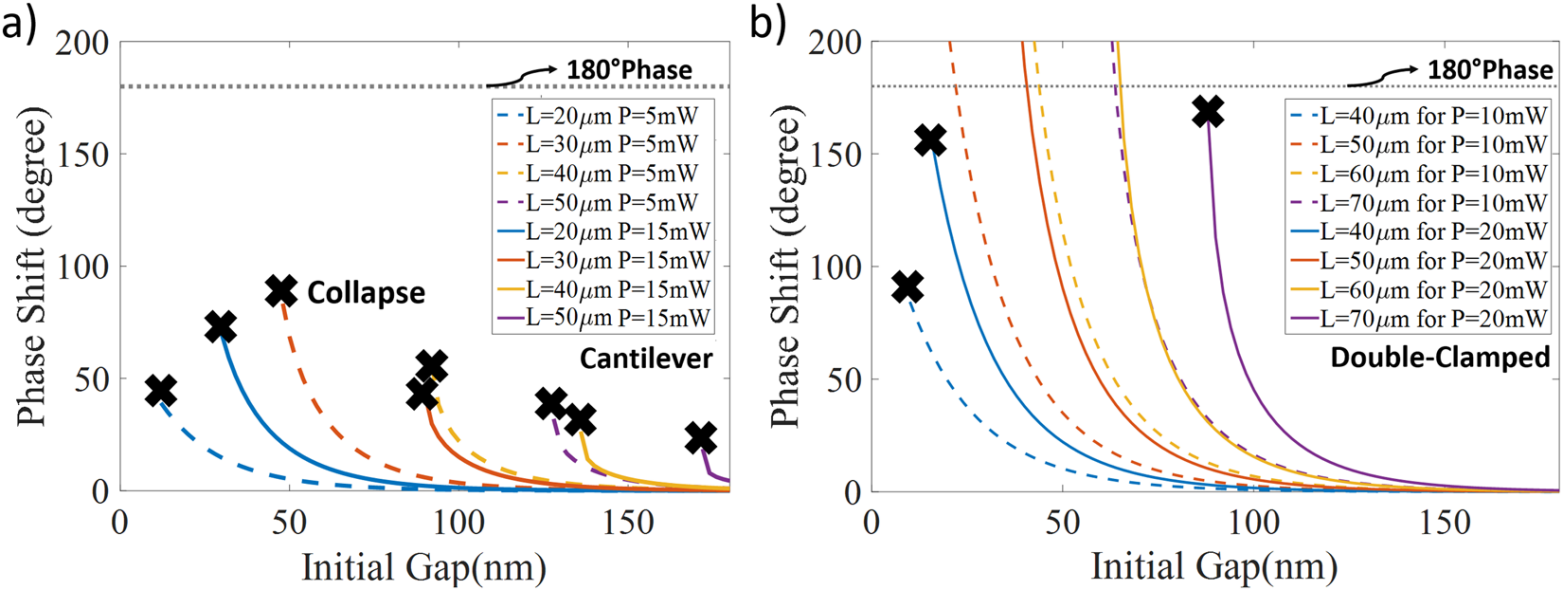
Generated phase values with respect to the initial gap. (**a)** The beam is cantilever (*P* = 5 mW & 15 mW and *l* = 20 → 50 µm). (**b)** The control waveguide is double-clamped (*P* = 10 mW & 20 mW and *l* = 40 → 70 µm). Required phase shift of 180° is represented with black dashed line, where the cross insets show collapse points.

For a clearer illustration of the data seen in Fig. 4, the phase values with respect to the initial separation for a specified input power is replotted in Fig. 5. Figure 5a demonstrates the results for the cantilever case where maximum generated phase just before collapse point decrease once the initial gap gets smaller or the interaction length gets longer. Following the idea of the formula introduced above, both smaller separation and longer device length will result in an increase in the displacement for the same power; hence, the physical contact of the beams is expected to occur easier for the devices with these properties. In addition, the maximum achievable phase difference with cantilever structure is ~80°, which is less than half of the required value for switching. Furthermore, as shown in the Fig. 5b, the double-clamped case also collapses before being able to provide 180° phase when the separation is smaller or the control beam is longer than a certain value.

Besides the geometrical properties, the material dependent parameters (effective index change with respect to the separation and Young’s modulus (E)) have also important roles in determining the beam displacement and the optical force. Gallium arsenide (GaAs) and silicon nitride (SiN), which are the other commonly used semiconductors for designing opto-mechanical devices, have refractive indices close to silicon. However, their Young’s modulus are quite different (E_Si_ = 131 GPa, E_SiN_ = 265 GPa, E_GaAs_ = 85.5 GPa) with a potential to influence the deflection behavior of the beams. However, simulations based on GaAs and SiN materials with the same device geometries showed that cantilever beam phase shifters are still inadequate to generate 180° phase shift (see Supplementary Information) for the other materials as well. Even though a higher Young’s modulus improves the strength of the beams for SiN, increased input power requirement limits the maximum phase difference possible with cantilevers. On the other hand, utilization of GaAs increases the mobility of beams further, which makes the beams more sensitive and harder to balance.

Since the double-clamped beams have been shown to be more stable in terms of the variation in the phase with respect to the input power as well as the physical strength, the rest of this section here focuses on analyzing the double-clamped structure. In particular, the fluctuations in the phase difference is quantified since the variations can disturb the intended device operation severely.

**Figure 6:**
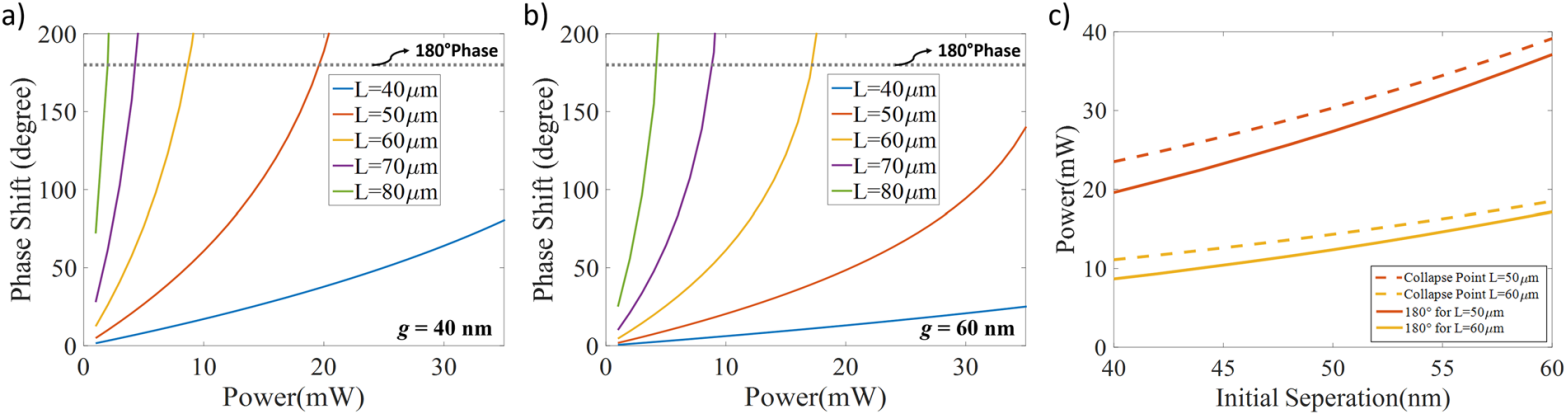
Phase generation values with respect to the input laser power are represented for initial separations of (**a)** 40 nm and (**b)** 60 nm, where 180° phase value is marked as a dashed line. (**c)** Power requirement for a 180° degree phase shift (solid lines) and physical stability edge (dashed lines) with respect to the initial separation. Double-clamped topology is utilized for all devices.

Figure 6a shows the generated phase shift for a double-clamped structure with 40 nm initial separation for a variety of different interaction lengths between 40 µm and 80 µm. While 1 mW variation in the input power results in ~21.6° phase difference for double-clamped geometry with 180° generated phase difference (*l* = 50 µm*, g = *40 nm*, P = *19.6 mW), this value raises to ~39.3° as device length increases to 60 µm (at a lower power level; *P* = 8.7 mW). As a result, the same input power generates more deflection (higher phase difference) for longer devices in the expense of making the system more sensitive to the power variation. The calculations in Fig. 6a have been repeated with an initial separation of 60 nm and the results are summarized in Fig. 6b. The clear and expected observation is that the structures with higher separation requires a higher deflection (i.e. more power: ~8.6 mW is required for *g* = 40 nm and 60 µm of device length while this value is ~17.1 mW for *g* = 60 nm) for the same waveguide length even though the 1 mW change sensitivity can be improved.

The results in Fig. 6 show that if the main design parameter is to have low sensitivity to input power variations, the phase shifter has to be either shorter or designed with a wider gap between the signal and the control waveguides. On the other hand, if the required power is the most critical value to be optimized, longer devices with smaller initial gaps are necessary. Furthermore, despite the current advanced technology, limitation coming from the fabrication techniques will also play a major role in selecting the waveguide dimensions due to the fact that smaller gaps and the longer released waveguides still have practical challenges. Thus, there is a design problem consisting of trade-offs between stability, low power and fabrication challenges. Double-clamped structures with separations larger than 40 nm and 40–60 µm long interaction lengths seem to be able to provide a decent performance together with minimized variations in the output.

Finally, Fig. 6c demonstrates the comparison between the operation conditions providing 180° phase difference and the situation where the two waveguides collapse for these structures. Operation conditions get closer to the stability edge with the increased initial separation due to the fact that higher required displacement makes system harder to balance. On the other hand, shorter devices have greater margin between collapse point and π phase generation point with ~2.5–3.7 mW input power range for ~45 nm initial separation (waveguide length of 50–60 µm).

## Conclusion

In summary, the stability of phase shifters with commonly used beam geometries, cantilever and double-clamped beams are investigated and the requirement for a precise iterative mathematical solution that includes the effect of force variations generated from the displacement along the beams is shown. A numerical formulation is offered for the maximum utilizable power as a function of initial separation and device length. With this approach, device characteristics in the case of physical variations during the fabrication is possible to predict. Significant deflections have been seem to be essential to generate considerable phase shift and required displacement of beams may lead to an unstable state for these structures especially for cantilever structures. Consequently, φ = 180° phase shift is shown to be theoretically impossible to achieve with the utilization of cantilever beams. On the other hand, a functional phase shifter can be fabricated by using the optimum physical properties for double-clamped structures. Detailed analysis towards the controllability of the phase shift besides the stability and phase generation range are also presented.

## Methods

An accurate technique to evaluate optically generated forces is utilizing Maxwell tensor method^43^. Alternatively, Response Theory of Optical Forces (RTOF)^44^ is proposed for the same purpose showing perfect agreement with Maxwell tensor method. Due to faster and convenient computation of RTOF method, it is more suitable for numerical analysis of opto-mechanical devices. Therefore, generated optical force is evaluated by^45^,

3 $$F(d)=\frac{LP}{c}\frac{\partial {n}_{eff}}{\partial d},$$

F represents the force, L device length and P launched power, where c stands for speed of light, *n*_*eff*_ for effective index and d for the separation. Then, in order to evaluate effect of continuous force exposed to the beam and waveguides, deflection values are determined by Euler-Bernoulli beam theory^42^,

4 $$\frac{{\partial }^{2}u}{\partial {z}^{2}}(EI\frac{{\partial }^{2}u}{\partial {z}^{2}})=\frac{LAE{t}^{2}}{12}\frac{{\partial }^{4}u}{\partial {z}^{4}}=F,$$

where u is deflection, E = 131 GPa is Young’s modulus of silicon, A is device cross-section area and t is thickness (500 nm for the waveguide, 250 nm for the beam). Utilization of Euler-Bernoulli beam theory requires two different initial conditions. Boundary conditions for fixed-ends of the waveguide and double-clamped and for a free-end of a cantilever are given in equations (5) and (6) respectively^45^.

5 $$u(0)=\frac{\partial u(0)}{\partial z}=u(l)=\frac{\partial u(l)}{\partial z}$$

6 $$u(0)=\frac{\partial u(0)}{\partial z}=\frac{{\partial }^{2}u(l)}{\partial {z}^{2}}=\frac{{\partial }^{3}u(l)}{\partial {z}^{3}}$$

Assuming that force in the Equation (4) is independent of z component of the Cartesian space, the following solutions can be concluded as a good approximation.

7 $$u(z)=\frac{0.5F}{AL{t}^{2}E}{z}^{4}-\frac{F}{A{t}^{2}E}{z}^{3}+\frac{0.5FL}{A{t}^{2}E}{z}^{2}$$

8 $$u(z)=\frac{0.5F}{AL{t}^{2}E}{z}^{4}-\frac{2F}{A{t}^{2}E}{z}^{3}+\frac{3FL}{A{t}^{2}E}{z}^{2}$$

Note that, the variations generated due to the initial assumption should be considered by iteratively solving the simplified equations.

Wavelength of both pump and signal are chosen to be 1550 nm and 1480 nm. The effect of laterally coupled light field to the effective index with respect to the separation and all optical properties are calculated with 3D finite element (FEM) simulations in COMSOL. Force and deflection values are determined by the in-house numerical tool, which iteratively solves the given equations.

### Data availability

All data generated and analyzed during this study are included in this published article (and its Supplementary Information files).

## Electronic supplementary material

Supplementary Information
