# Supplementary material for: Stability Formulation for Integrated Opto-mechanic Phase Shifters
Source: Sci Rep. 2018 Jan 31;8:1937. doi: 10.1038/s41598-018-20405-1 (PMC5792635; doi:10.1038/s41598-018-20405-1)
Supplement: Supplementary file 1 — Supplementary Information [file 41598_2018_20405_MOESM1_ESM.pdf]

Stability Formulation for Integrated Opto-mechanic Phase Shifters

Yigit Ozer [a] and Serdar Kocaman[*a]

[a] Electrical and Electronics Engineering Department, Middle East Technical University, Ankara, 06800, Turkey

Supplementary Information

Analysis of proposed formulation on different opto-mechanical phase shifter design

The proposed maximum utilizable power formulation is utilized for another opto-mechanical phase shifter design, which is taken from previous study [9]. This phase shifter consists of waveguide width of 400 nm and control beam width of 200 nm, where the thickness is 1 µm. Fig. S1.a represents the collapse points for cantilever structure and Fig. S1.b shows these points for double-clamped beam case.

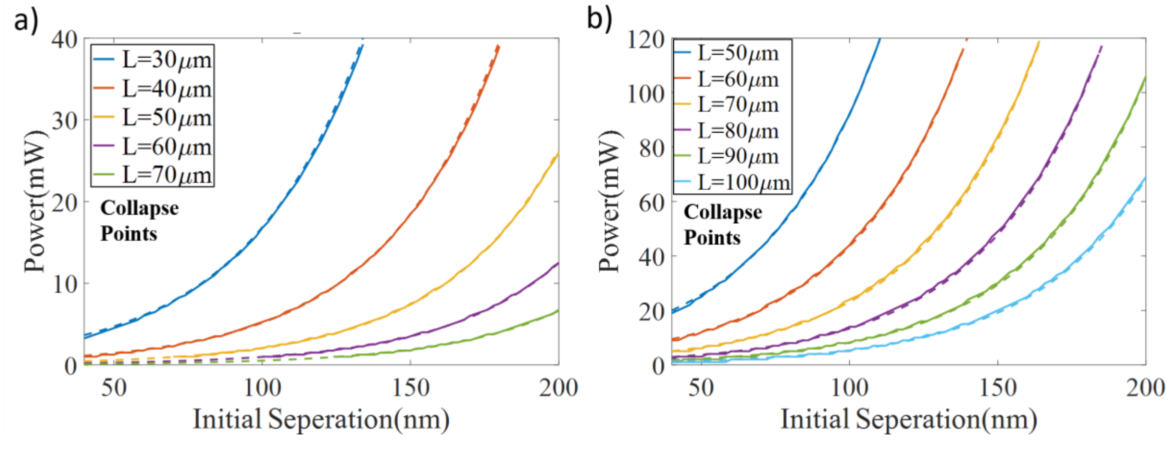

Figure S1: Stability condition for different phase shifter designs. The simulated collapse points are represented with solid lines where proposed formulation results are shown as dashed lines for **a)** cantilever and **b)** double-clamped beams. The region under each curve represents the conditions.

* Corresponding author: skocaman@metu.edu.tr

For this design, the constants of the proposed formulation (Equation 1) determining the power dependency to the device length and initial separation (B = 0.0255 and C = 4.109) are the same as in the case for previous structures [10]. Only change in our analysis is the constant A, which is proportionality constant in Equation 1. This constant changed as $A = 2.57\text{x}10^{10}$ for cantilever beams and $A = 1.149\text{x}10^{12}$ for double-clamped beam cases for the new structure. This result proves that proposed mathematical approach is accurate in representing the physical device parameter dependencies.

Phase generation is also simulated for these designs and the results are represented in Fig. S2. Similar to the previous design [10], cantilever beams are unable to generate required phase shift for the switching operation since they collapse before significant phase generation. Even for the optimized cantilever structure, the maximum achievable phase shift is below 80°.

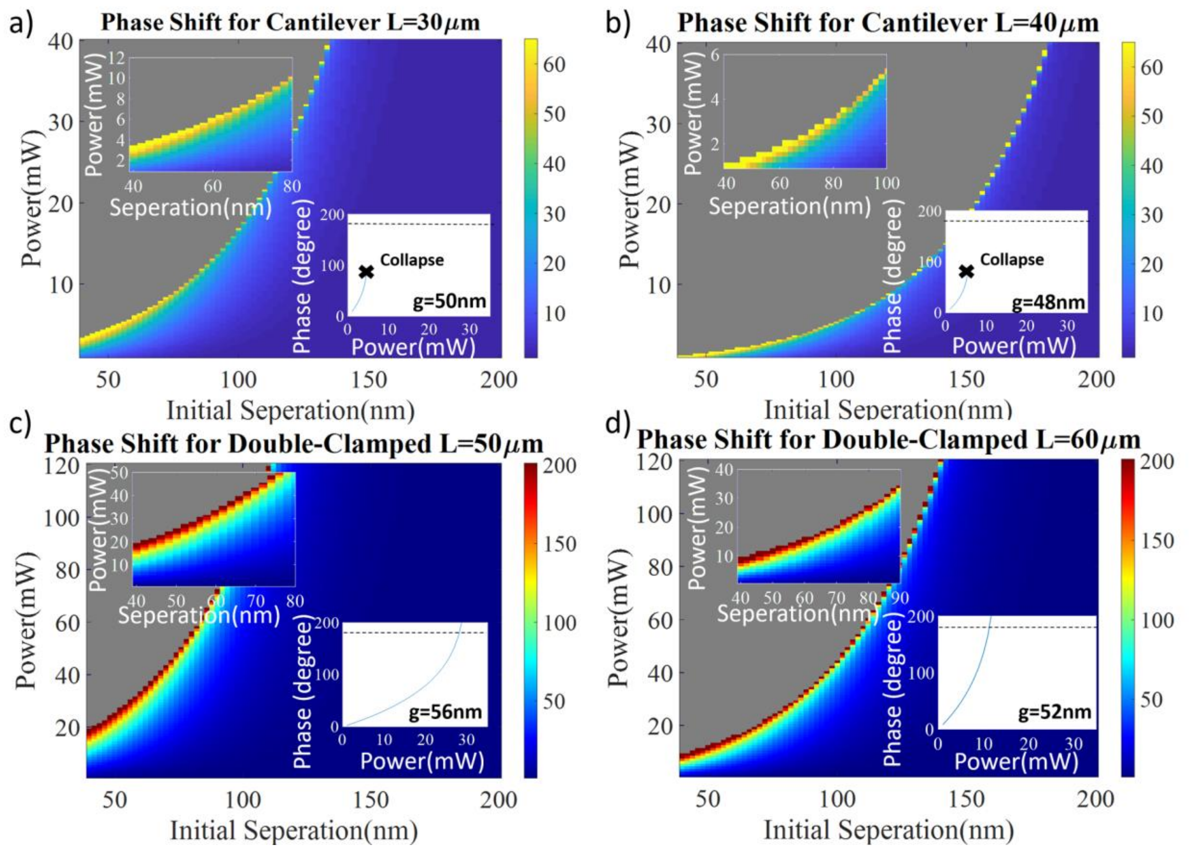

Figure S2: Generated phase shift (in color) with respect to the power and initial separation values. Physically unstable conditions are represented in gray color. The insets show zoom of the ideal operation regions and generated phase values with respect to the input power for specified initial

separation. **a), b)** Phase generation of cantilever beam structures for 30μm and 40μm of device lengths. **c), d)** Generated phase difference for double-clamped beams of 50μm and 60μm device lengths.

The behavior of the opto-mechanical phase shifters are similar in phase generation as well as the stability condition. Fig. S2 and Fig. S3 show the phase generation for various conditions and provide similar results representing the unsuitability of cantilever beams in opto-mechanical phase shifters.

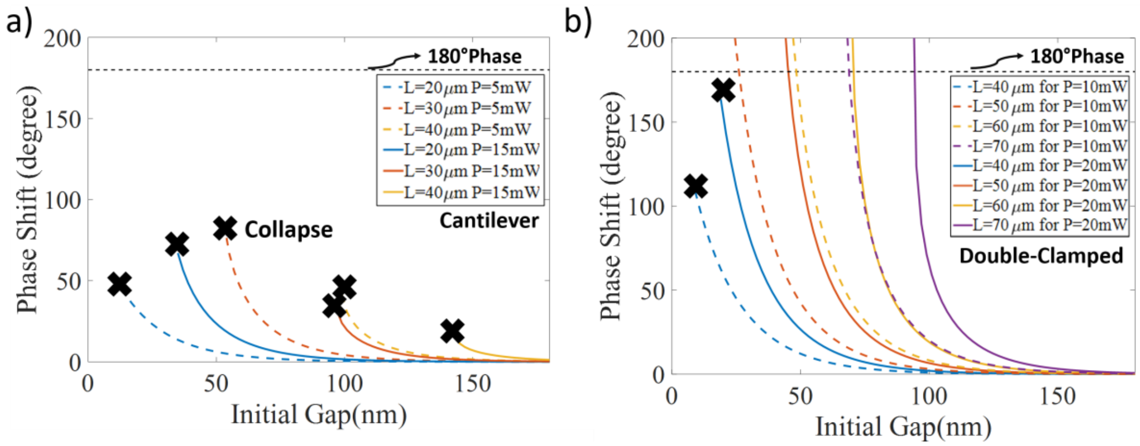

Figure S3: Phase generation with respect to the initial gap. a) The beam is cantilever (P = 5 mW & 15 mW and l= 20→50 μm). **b)** The control waveguide is double-clamped (P = 10 mW & 20 mW and l= 40→70 μm). Required phase shift of 180° is represented with black dashed line, where the cross insets show collapse points.

As shown in Fig. S4, double-clamped structures are able to provide successful operation. Considering the power requirement and fabrication challenges, optimum operation regime is the conditions where initial separation is ~45 nm and device length is between 50 μm – 60 μm. As clearly shown in Fig. S4.a and S4.b, a larger initial separation increases the required input power for switching operation as expected.

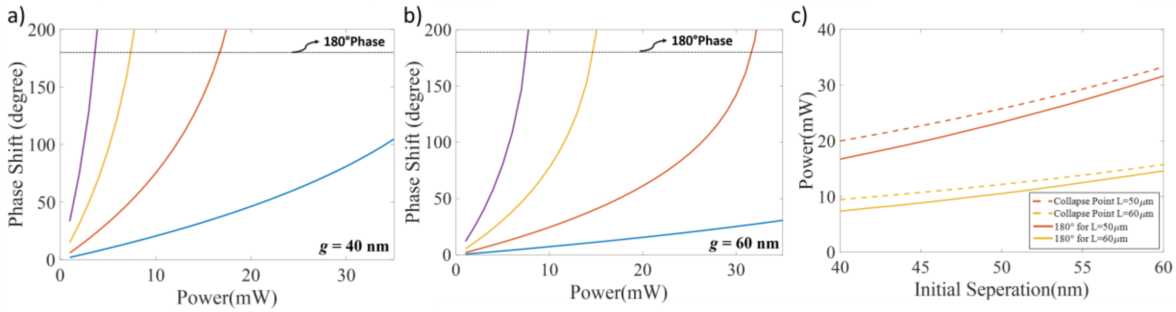

Figure S4: Phase generation values with respect to the input laser power are represented for initial separations of **a)** 40 nm and **b)** 60 nm, where 180° phase value is marked as a dashed line. **c)** Power requirement for a 180° degree phase shift (solid lines) and physical stability edge (dashed lines) with respect to the initial separation. Double-clamped topology is utilized for all devices.

Fig. S4.c represents the collapse points and the 180° phase generation points together. According to the data, the power margins between collapse points and optimum operation conditions are ~2.2-3.4 mW, which is very close to the case for the previous design [10].

Analysis of cantilever beams with other semiconductor materials

The opto-mechanical phase shifters with cantilever beams discussed in the main text are also implemented by using silicon nitride and gallium arsenide. Even though the Young's modulus of these materials have a direct influence on the deflection value, the maximum achievable phase is still insufficient for switching operation for both lower and higher Young's modulus values (E_{Si}=131 GPa, E_{SiN}=265 GPa, E_{GaAs}=85.5 GPa). Fig. S5 represents the achieved phase shift with respect to the separation for various phase shifter designs and input power values.

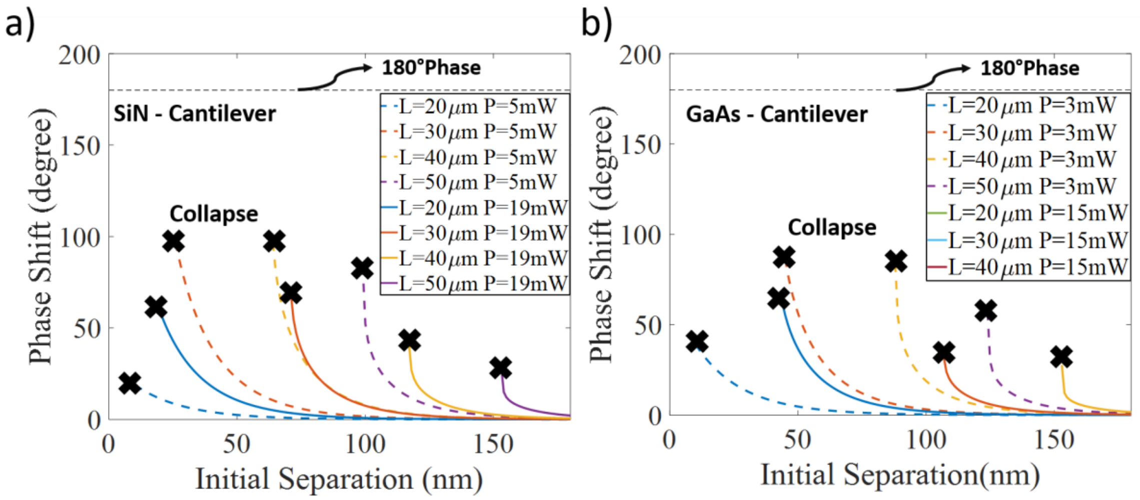

Figure S5: Phase generation with respect to the initial gap. a) The phase shifter design with SiN cantilever beam (P = 5 mW & 19 mW and l = 20→50 μm). **b)** GaAs based cantilever design (P = 3 mW & 15 mW and l = 20→50 μm). Required phase shift of 180° is represented with black dashed line, where the cross insets show collapse points.

For device geometry considered here, optimum input power that generates the highest possible phase shift changes with the Young's Modulus due to its strong effect on deflection. Therefore, phase generation is investigated for a wide range of Young's Modulus and the input power where the length of phase shifter with cantilever beam is 30 μm and the initial separation is 60 nm (Fig. S6). The results showed that the maximum phase shift value does not considerably change with the material properties; however, the input power values that give the highest possible phase difference are different for each material.

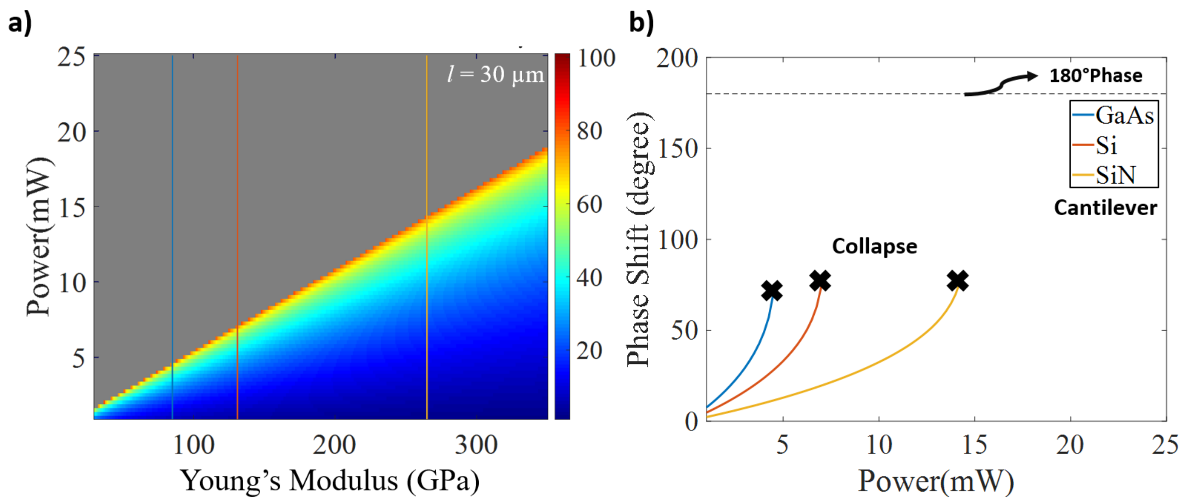

Figure S6: a) Generated phase shift (in color) with respect to the power and Young's Modulus for a cantilever beam with l = 30 µm and g = 60 nm. Unstable conditions are represented in gray color. The blue, orange and yellow lines represent GaAs, Si and SiN materials respectively. **b)** Phase generation of cantilever beam structures based on GaAs, Si and SiN materials with respect to the input power.
